# Profile of esophageal squamous cell carcinoma mutations in Brazilian patients

**DOI:** 10.1038/s41598-021-00208-7

**Published:** 2021-10-18

**Authors:** Fernanda Franco Munari, Wellington dos Santos, Adriane Feijó Evangelista, Ana Carolina Carvalho, Paula Aguiar Pastrez, Diego Bugatti, Durval R. Wohnrath, Cristovam Scapulatempo-Neto, Denise Peixoto Guimarães, Adhemar Longatto-Filho, Rui Manuel Reis

**Affiliations:** 1 Molecular Oncology Research Center, Barretos Cancer Hospital, Antenor Duarte Villela, 1331, Barretos, São Paulo 14784 400 Brazil; 2 Department of Upper Digestive, Barretos Cancer Hospital, Barretos, Brazil; 3 Department of Pathology, Barretos Cancer Hospital, Barretos, Brazil; 4 Department of Endoscopy, Barretos Cancer Hospital, Barretos, Brazil; 5 Medical Laboratory of Medical Investigation (LIM) 14, Department of Pathology, Medical School, University of São Paulo, São Paulo, Brazil; 6 Life and Health Sciences Research Institute (ICVS), School of Medicine, University of Minho, Braga, Portugal; 7 ICVS/3B’s-PT Government Associate Laboratory, Braga, Guimarães, Portugal

**Keywords:** Cancer genomics, Gastrointestinal cancer

## Abstract

Esophageal cancer is an aggressive tumor that has a high rate of incidence and mortality worldwide. It is the 10th most frequent type in Brazil, being squamous cell carcinoma (ESCC) the predominant subtype. There is currently an incessant search to identify the frequently altered genes associated with esophageal squamous cell carcinoma biology that could be druggable. This study aimed to analyze the somatic mutation profile of a large panel of cancer-related genes in Brazilian ESCC. In a series of 46 ESCC diagnoses at Barretos Cancer Hospital, DNA isolated from paired fresh-frozen and blood tissue, a panel of 150 cancer-related genes was analyzed by next-generation sequencing. The genes with the highest frequency of mutations were *TP53* (39/46, 84.8%), followed by *NOTCH1* (7/46, 15.2%), *NFE2L2* (5/46, 10.8%), *RB1* (3/46, 6.5%), *PTEN* (3/46, 6.5%), *CDKN2A* (3/46, 6.5%), *PTCH1* (2/46, 4.3%) and *PIK3CA* (2/46, 4.3%). There was no significant association between molecular and patients’ clinicopathological features. Applying an evolutionary action score of p53 (EAp53), we observed that 14 (35.9%) *TP53* mutations were classified as high-risk, yet no association with overall survival was observed. Concluding, this the largest mutation profile of Brazilian ESCC patients, which helps in the elucidation of the major cancer-related genes in this population.

## Introduction

Esophageal cancer is an aggressive tumor with high incidence and mortality rates^1,2^. Worldwide estimates show that esophageal cancer is the seventh most common type, with around 572,000 esophageal cancer cases in 2018^1,2^. In Brazil, esophageal cancer is among the ten most common tumor types, ranking 6th among men and 15th among women^3^. According to the National Cancer Institute (INCA), for the year 2020–2021, it is estimated that about 11390 new cases, 8690 in men and 2700 in women, also presenting mortality rates close to the incidence rates^3^. Esophageal cancer's poor prognosis is due to a lack of specific symptoms in the early stages of the disease and late diagnosis, with less than 20% of cases showing an overall five-year survival^4,5^. However, when diagnosed early, the five-year survival rate increases to 80–90%^5^.

Histologically, 90% of cases are esophageal squamous cell carcinoma (ESCC) and 12% of adenocarcinoma (EADC)^5^. The highest incidence of ESCC occurs in northern Iran to central north China and in developing countries such as Brazil, whereas EADC occurs more frequently in developed countries^3,5–7^. ESCC affects the middle third of the esophagus and shares several characteristics with head and neck squamous cell carcinoma^5,8^. The main risk factors are tobacco and alcohol consumption (especially in combination), hot food and beverage intake^5,9,10^.

Recent extensive comprehensive molecular studies determine the genomic landscape of ESCC^11–17^. The *TP53* was the most frequent mutated gene observed all over the studies, and genes involved in other essential cancer pathways, such as the cell cycle, PI3K, and NOTCH pathways, were also reported^11–17^. Of note, the integrative TCGA consortium identified three molecular subtypes: ESCC1, associated with alterations in the NRF2 pathway, which regulates adaptation to oxidative stressors, and gene expression profile resembles lung cancer and head and neck squamous carcinoma; ESCC2, with higher *NOTCH1* mutation rate and deregulation of apoptosis: and ESCC3 with upregulation of the PI3K pathway and lower *TP53* mutation rate^17^. Interestingly, the three ESCC subtypes tended geographic associations, being the ESCC1 more prevalent in the Asian population, the ESCC2 in Eastern European and South American (Brazil), and the ESCC3 observed only in North America^17^. The characterization of genes involved in ESCC tumorigenesis is crucial to understand its biology and help identify putative cancer biomarkers and targeted therapies^13^.

The molecular profile of ESCC is mainly unknown in Brazil, being mainly restricted to *TP53* mutation status^7,18^. Therefore, this study aimed to identify the somatic mutational profile of Brazilian ESCC patients by analyzing the entire coding region of a panel of 150 cancer-related genes by next-generation sequencing.

## Results

### Description of the clinicopathological features

The summary of clinicopathological features of the esophageal squamous cell carcinoma studied is reported in Table 1. We observed that the population was primarily composed of men (39 cases, 84.8%) with an average age of 60 years (minimum 39 years and maximum 77 years); most of them consumed alcohol (37 cases, 82.2%) and tobacco (36 cases, 80.0%). The most used these substances in combination (32 cases, 71.1%). Most patients presented moderately differentiated tumors (26 cases, 59.1%), whose clinical stage was most advanced III and IV (36 cases, 87.8%). Patients' median survival concerning the tumor diagnosis data until the last information (death or follow-up) was 9.40 months.

**Table 1:** Clinical-pathological features of esophageal squamous cell carcinoma patients.

Variable	ESCC (n = 46)		
	Category	n	%
Age (years)	Mean (SD)	60	–
	Min–Max	39–77	–
Gender	Female	7	15.2
	Male	39	84.8
Alcohol consumption	No	8	17.4
	Yes	37	82.6
	Missing	1	–
Tobacco consumption	No	9	19.6
	Yes	36	80.4
	Missing	1	–
Tobacco and alcohol in combination	No	13	28.3
	Yes	32	71.7
	Missing	1	–
Tumor differentiation	Little	14	31.8
	Moderate	26	59.1
	Well	4	9.1
	Missing	2	–
TNM Staging*	I e II	5	10.8
	III e IV	36	89.2
	Missing	5	–
Life status	Alive (without cancer)	1	2.2
	Alive (with cancer)	6	13.0
	Dead (by cancer)	39	84.8

*TNM 7 edition staging.

*n* number of cases, *ESCC* esophageal squamous cell carcinoma.

### Description of the mutation profile

**Figure 1:**
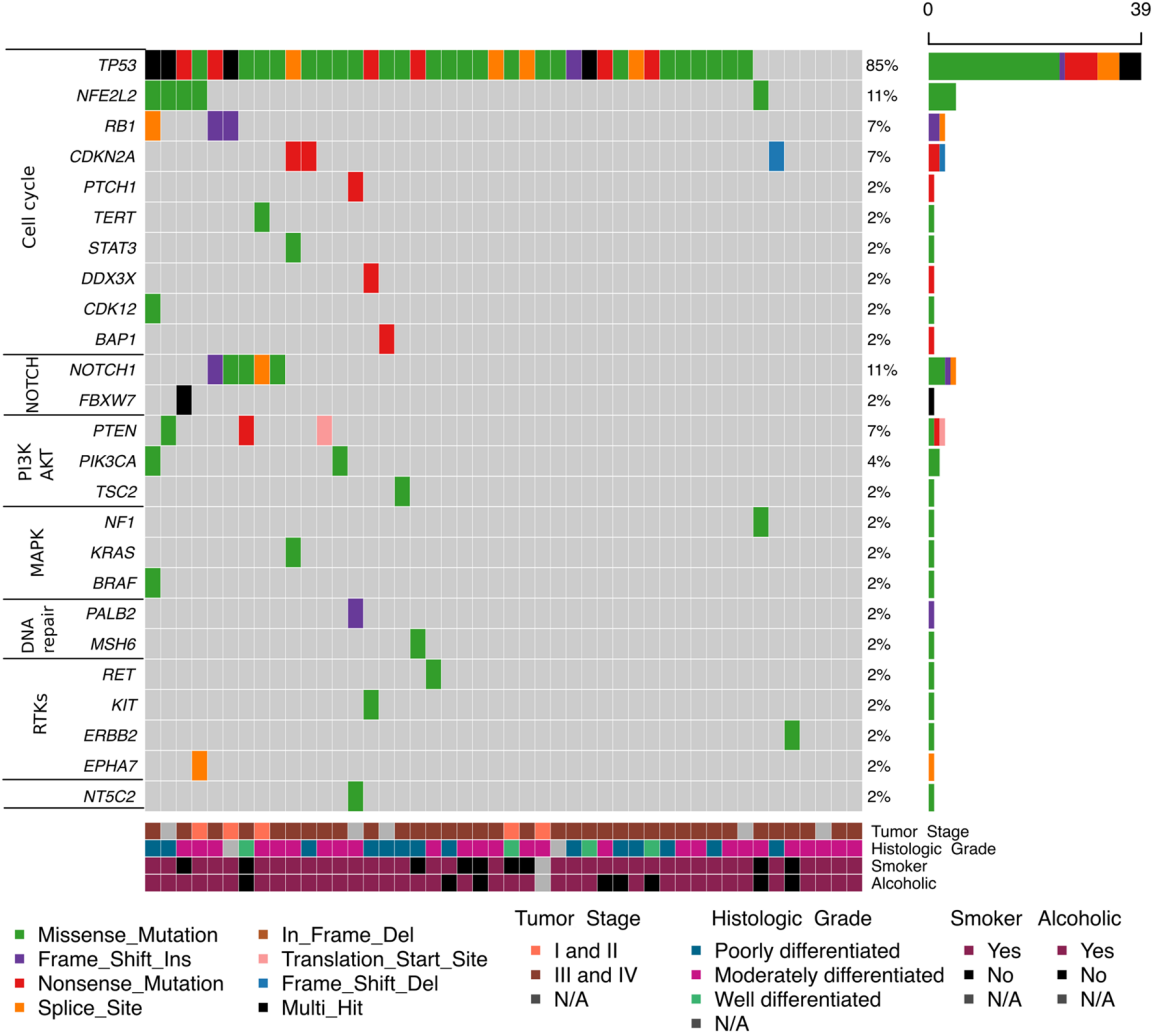
Oncoplot of the distribution of mutations found in cases of esophageal squamous cell carcinoma. The upper graph shows the mutation frequency for each tumor sample. The left graph shows the frequency of samples with mutations. The central graph shows the types of mutations in each tumor sample. The lower part of the figure shows the clinical-pathological data (tumor stage in the diagnosis and histological differentiation) of each sample.

We sequenced the whole coding region of 150 cancer-related genes in 46 cases of esophageal squamous cell carcinoma. The mean read depth of sequencing was 911 × per gene and 310.9 × per variant. We found a mean of 1.9 driver mutations per patient (range 0–7), and we identified at least one driver somatic variant in 42 tumor samples. Driver mutations in single genes were found in 41.3% of tumors (19/46), whereas 26.1% (12/46) showed driver mutations in two genes, 17.4% (8/46) in three genes, 6.5% (3/46) in four or more genes and 8.7% (4/46) showed driver mutations in none of the genes analyzed. In total, 25 genes were found to harbor driver somatic mutations (Fig. 1). A complete list of variants (missense, frameshift, nonsense, in-frame, and splice mutations) identified is presented in Supplementary Table S1.

The gene with the highest frequency of driver mutations was *TP53* (39 cases, 85%), followed by *NOTCH1* (5 cases, 11%), *NFE2L2* (5 cases, 11%)*, RB1* (3 cases, 7%), *PTEN* (3 cases, 7%), *CDKN2A* (3 cases, 7%), *PTCH1* (1 case, 2%) and *PIK3CA* (2 cases, 4%) (Fig. 1). Below, we describe in more detail the most affected genes and pathways.

### Genes involved in the cell cycle

Of the 25 genes with driver mutations identified, ten were involved in the cell cycle (Fig. 1; Supplementary Table S1). Overall, 41 (89.1%) cases showed mutation of this crucial cancer pathway.

**Figure 2:**
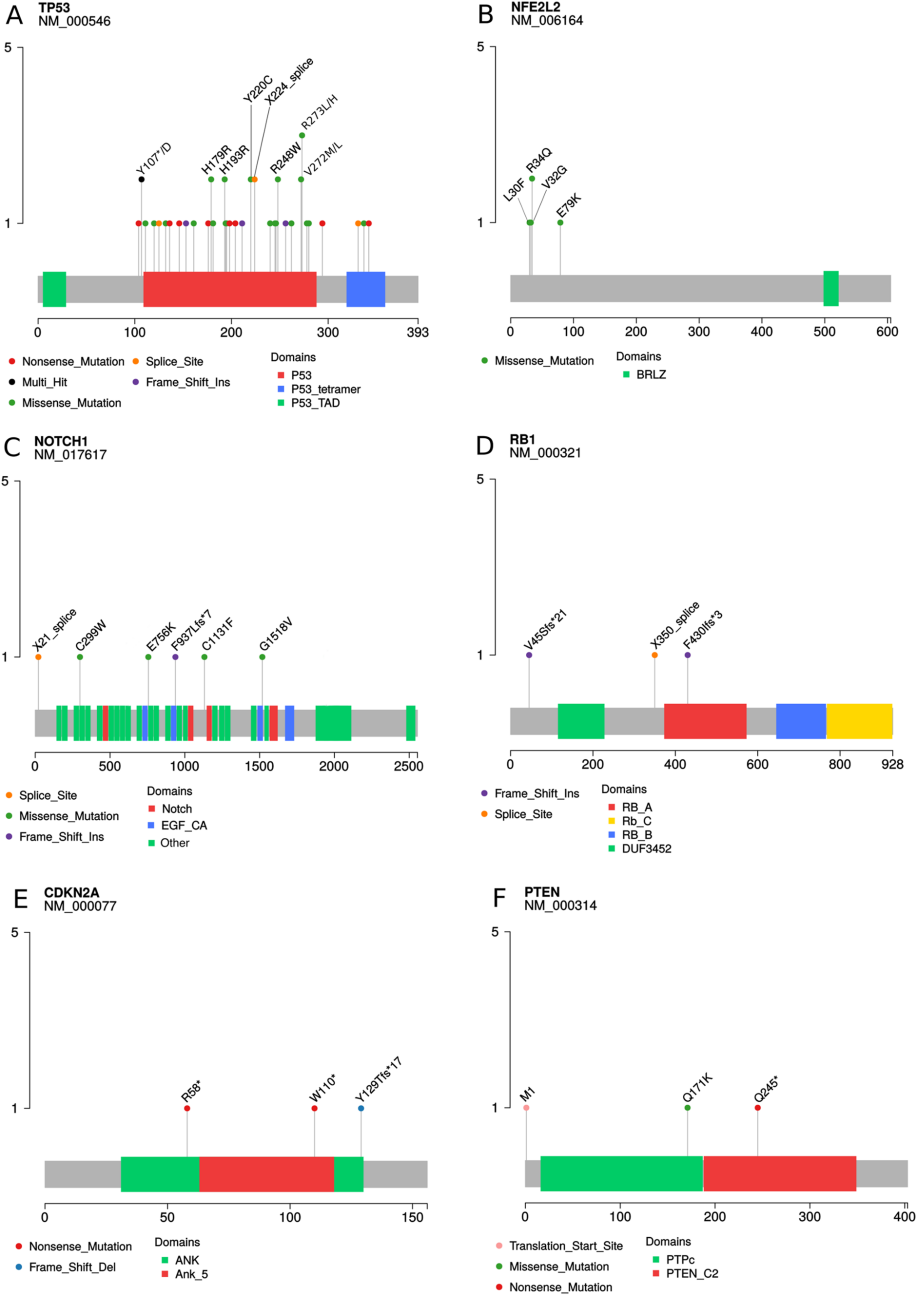
Lollipop plot of the genes with the highest frequency of mutations.

A total of 44 somatic *TP53* mutations were identified in 39 tumors (85%) (Fig. 1; Supplementary Table S1). The mutations were 28 missense, 9 nonsense, 4 splice sites, and 3 frameshift. The most frequent changes were p.His193Arg, p.His179Arg, p.Arg248Trp, p.Arg273Leu, p.Tyr107Asp, and p.Tyr220Cys, all of them previously reported. We also identified two changes (p.Thr211AsnfsTer5 and p.Thr256HisfsTer8) that have not been previously identified. (Fig. 2A).

Five cases (11%) showed somatic mutations in the *NFE2L2* gene (Figs. 1, 2 and Supplementary Table S1). The mutations were all missense, being p.Arg34Gln and p.Val32Gly in 2 cases, and p.Glu79Lys and p.Leu30Phe, which was present in one case each (Fig. 2B). Three tumors (7%) exhibited somatic changes in the *RB1* gene (Fig. 1; Supplementary Table S1). The mutations were two frameshifts and one splice site (Fig. 2D). The *CDKN2A* gene was mutated in 3 cases (7%) (Fig. 1; Supplementary Table S1). The types of changes identified in the *CDKN2A* gene were two nonsense and one frameshift (Fig. 2E). We also observed the *PTCH1* gene's somatic mutation in two tumors (2%) (Fig. 1; Supplementary Table S1). The types of changes identified were one missense and one nonsense. Moreover, we observed mutations in *TERT, STAT3, DDX3X, CDK12*, and *BAP1* genes in one case each (Supplementary Table S1).

### *NOTCH* signaling pathway alterations

The Notch signaling pathway involving the genes *NOTCH1* and *FBXW7* was altered in 6 cases (13%) (Fig. 1; Supplementary Table S1). The most affected gene was *NOTCH1* (11%), and the types of changes identified were 3 missense, one frameshift, and one splice site (Fig. 2C). Only one missense mutation was identified in FBXW7.

### PIK3-AKT signaling pathway alterations

The PIK3-AKT signaling pathway involving the genes *PTEN*, *PIK3CA*, and *TSC2* was altered in 6 (13%) cases (Fig. 1; Supplementary Table S1).

*PTEN* was the gene with the most mutations identified with alterations in 3 cases (7%). The types of changes identified were one missense, one nonsense, and one translocation (Fig. 2F). Mutations in the *PIK3CA* gene were identified in 2 cases (4%), at hotspot codons p.Glu542Lys and p.Glu545Lys in one case each. The *TSC2* gene was also mutated in one case (2%), and the alteration was a p.Thr831Met.

### MAPK signaling pathway alterations

The MAPK signaling pathway, with genes *KRAS*, *NF1*, and *BRAF*, was altered in 3 ESCC (7%) (Fig. 1; Supplementary Table S1). All three genes were changed in one case each (2%). All changes were missense type. The type of alterations in the *KRAS* gene was the p.Gly12Ser; in the *NF1* gene, it was p.Pro2115Leu, and in the *BRAF* gene, it was p.Arg260Cys, outside the known hotspot V600 region**.**

### Genes involved in the DNA repair

Of the 25 mutated genes, only 2 (*MSH6* and *PALB2*) were involved in the DNA repair (Fig. 1; Supplementary Table S1).

### RTKs signaling pathway alterations

All four genes of the RTKs signaling pathway had a mutation each (2%). The *RET* gene is the missense p.Arg1050Gln mutation; the *EPHA7* gene harbor the splice site p.Val777Leu mutation and the *ERBB2* gene have a missense mutation. In the *KIT* gene, the only mutation was for p.Val825Gly.

### Classification of *TP53* mutations

*TP53* mutations were further classified using the evolutionary action score of p53 (EAp53), which considers only missense mutations such as “low-risk” and “high-risk”^19^. Of the 23 mutations, we observe 14/23 (60%) as high-risk and 9 /23 (40%) as low-risk (Table 2). A second classification was done following Poeta et al., which categorize mutations as “disruptive” and “non-disruptive”^20,21^, and we observed 21/39 (54%) as disruptive and 18/39 (46%) as non-disruptive (Table 2).

**Table 2:** Classification of *TP53* mutations.

Variants	n (%)	Mutation type	Evolutionary action score of p53 (EAp53)		Disruptive/non-disruptive^c^
			EA score^a^	Risk in HNC^b^	
H193R	2 (4.3%)	Missense	85.96	High	Non-disruptive
H179R	2 (4.3%)	Missense	81.91	High	Non-disruptive
R273L	2 (4.3%)	Missense	87.45	High	Non-disruptive
Y220C	2 (4.3%)	Missense	72.52	Low	Non-disruptive
R248W	2 (4.3%)	Missense	84.11	High	Disruptive
P278L	1 (2.2%)	Missense	93.52	High	Non-disruptive
Y107D	1 (2.2%)	Missense	71.68	Low	Non-disruptive
A161D	1 (2.2%)	Missense	89.88	High	Non-disruptive
G245V	1 (2.2%)	Missense	98.74	High	Non-disruptive
G262V	1 (2.2%)	Missense	88.02	High	Non-disruptive
I195N	1 (2.2%)	Missense	87.79	High	Disruptive
K120N	1 (2.2%)	Missense	78.33	High	Non-disruptive
K132M	1 (2.2%)	Missense	95.30	High	Non-disruptive
L111Q	1 (2.2%)	Missense	74.95	Low	Non-disruptive
L194H	1 (2.2%)	Missense	83.64	High	Disruptive
M246V	1 (2.2%)	Missense	66.21	Low	Non-disruptive
R181P	1 (2.2%)	Missense	63.02	Low	Disruptive
R273H	1 (2.2%)	Missense	66.12	Low	Non-disruptive
R280I	1 (2.2%)	Missense	97.49	High	Non-disruptive
R337C	1 (2.2%)	Missense	63.41	Low	Non-disruptive
S240R	1 (2.2%)	Missense	80.92	High	Disruptive
V272L	1 (2.2%)	Missense	59.98	Low	Non-disruptive
V272M	1 (2.2%)	Missense	63.49	Low	Non-disruptive
T211NfsTer5	1 (2.2%)	Frameshift	NA	NA	Disruptive
P153AfsTer28	1 (2.2%)	Frameshift	NA	NA	Disruptive
T256HfsTer8	1 (2.2%)	Frameshift	NA	NA	Disruptive
E198Ter	1 (2.2%)	Nonsense	NA	NA	Disruptive
Q136Ter	1 (2.2%)	Nonsense	NA	NA	Disruptive
R342Ter	1 (2.2%)	Nonsense	NA	NA	Disruptive
E294Ter	1 (2.2%)	Nonsense	NA	NA	Disruptive
Q104Ter	1 (2.2%)	Nonsense	NA	NA	Disruptive
C176Ter	1 (2.2%)	Nonsense	NA	NA	Disruptive
W146Ter	1 (2.2%)	Nonsense	NA	NA	Disruptive
E204Ter	1 (2.2%)	Nonsense	NA	NA	Disruptive
Y107Ter	1 (2.2%)	Nonsense	NA	NA	Disruptive

*NA* not applicable, *n* number of cases.

^a^ http://mammoth.bcm.tmc.edu/cgi-bin/panos/EAp53.cgi.

^b^Neskey et al. Evolutionary Action Score of *TP53* Identifies High-Risk Mutations Associated with Decreased Survival and Increased Distant Metastases in Head and Neck Cancer, Cancer Res. 2015 Apr 1;75(7):1527–36; *n* number of variants.

^c^Poeta et al. TP53 mutations and survival in squamous-cell carcinoma of the head and neck. N Engl J Med 357, 2552–2561, https://doi.org/10.1056/NEJMoa073770 (2007).

### Association of mutation profile and clinicopathological features

Next, we performed an analysis of the association between clinicopathological features and mutation status. Due to the small number of cases analyzed, this analysis was performed only for genes harboring more than 10% of mutations, namely *TP53, NFE2L2,* and *NOTCH1* (Table 3). No significant association was found (Table 3). We also analyzed the association of *TP53* categories of (EAp53 risk score and disruptive and non-disruptive) with patients’ clinicopathological features, yet, no significant association was found (Table 4).

**Table 3:** Association between patients’ epidemiological and clinicopathological features with the *TP53, NFE2L2,* and *NOTCH1* status.

Variable	Category	ESCC (n = 46)														
		*TP53* gene					*NFE2L2* gene					*NOTCH1* gene				
		MUT (n = 39)		WT (n = 7)		*p*-value	MUT (n = 5)		WT (n = 41)		*p*-value	MUT (n = 5)		WT (n = 41)		*p*-value
		n	%	n	%		N	%	n	%		n	%	n	%	
Age (years)	Mean	59	–	57	–	0.597	65	–	58	–	0.205	62	–	59	–	0.495
	Min–Max	39–75	–	41–77	–		52–72	–	39–77	–		52–75	–	39–77	–	
Gender	Female	6	15.4	1	14.3	1.000	1	20.0	6	14.6	1.000	0	0.0	7	17.1	
	Male	33	84.6	6	85.7		4	80.0	35	85.4		5	100	34	82.9	
	Missing	0				–	0				–	0				
Alcohol consumption	No	6	15.8	2	28.6	0.590	1	20.0	7	17.5	1.000	1	20.0	7	17.5	1.000
	Yes	32	84.2	5	71.4		4	80.0	33	82.5		4	80.0	33	82.2	
	Missing	1				–	1				–	1				
Tobacco consumption	No	7	18.4	2	28.6	0.614	2	40.0	7	17.5	1.000	1	20.0	8	20.0	1.000
	Yes	31	81.6	5	71.4		3	60.0	33	82.5		4	80.0	32	80.0	
	Missing	1				–	1				–	45				
Tobacco and alcohol in combination	No	11	28.9	2	28.6	1.000	2	40.0	11	27.5	0.617	1	20.0	12	30.0	1.000
	Yes	27	71.1	5	71.4		3	60.0	29	72.5		4	80.0	28	70.0	
	Missing	1				–	1				–	1				
Tumor differentiation	Little	13	35.1	1	14.3	0.291	2	40.0	12	30.8	1.000	0	0.0	14	35.0	0.270
	Moderate	20	54.1	6	85.7		3	60.0	23	59.0		3	75.0	23	57.5	
	Well	4	10.8	0	0.0		0	0.0	4	10.3		1	25.0	3	7.5	
	Missing	2				–	2				–	2				
TNM Staging*	I e II	5	14.3	0	0.0	1.000	1	25.0	4	10.8	0.418	2	40.0	3	8.3	0.104
	III e IV	30	85.7	6	100.0		3	75.0	33	89.2		3	60.0	33	91.7	
	Missing	5				–	5				–	5				

*TNM 7 edition staging.

*n* number of cases.

*ESCC* esophageal squamous cell carcinoma.

*MUT* mutated.

*WT* wild type.

**Table 4:** Association between patients’ clinicopathological features with the classification of *TP53* mutations.

Variable	Category	EAp53 status^a,b^				*p*-value	Disruptive/Non-disruptive classification^c^				*p*-value
		High		Low			Disruptive		Non-disruptive		
		n	%	N	%		n	%	n	%	
Age (years)	Mean (SD)	58 (10.35)	–	64 (7.38)	–	0.196	60 (8.99)	–	60 (10.08)	–	0.660
	Min–Max	39–73	–	53–75	–		44–75	–	39–73	–	
	Missing	0									
Gender	Female	4	22.2	0	0.0	0.268	4	66.4	2	33.3	0.667
	Male	14	77.8	9	100.0		17	51.5	16	48.5	
	Missing	0					0				
Alcohol consumption	No	3	17.6	1	11.1	1.000	3	42.9	4	57.1	0.682
	Yes	14	82.4	8	88.9		18	56.3	14	43.8	
	Missing	1					0				
Tobacco consumption	No	3	17.6	1	11.1	1.000	5	62.5	3	37.5	0.702
	Yes	14	82.4	8	88.9		16	51.6	15	48.4	
	Missing	1					0				
Tobacco and alcohol in combination	No	5	29.4	1	11.1	0.380	7	58.3	5	41.7	0.742
	Yes	12	70.6	8	88.9		14	51.9	13	48.1	
	Missing	1					0				
Tumor differentiation	Little	4	22.2	4	57.1	0.251	7	53.8	6	46.2	1.000
	Moderate	12	66.7	2	28.6		11	55.0	9	45.0	
	Well	2	11.1	1	14.3		2	50.0	2	50.0	
	Missing	2					2				
TNM Staging*	I e II	3	18.8	2	25.0	1.000	1	25.0	3	75.0	0.312
	III e IV	13	81.3	6	75.0		18	58.1	13	41.9	
	Missing	3					4				

*TNM 7 edition staging; n – number of cases.

^a^ http://mammoth.bcm.tmc.edu/cgi-bin/panos/EAp53.cgi.

^b^Neskey et al. Evolutionary Action Score of *TP53* Identifies High-Risk Mutations Associated with Decreased Survival and Increased Distant Metastases in Head and Neck Cancer, Cancer Res. 2015 Apr 1;75(7):1527–36.

*n* number of variants.

^c^Poeta et al. TP53 mutations and survival in squamous-cell carcinoma of the head and neck. N Engl J Med 357, 2552–2561, https://doi.org/10.1056/NEJMoa073770 (2007).

**Figure 3:**
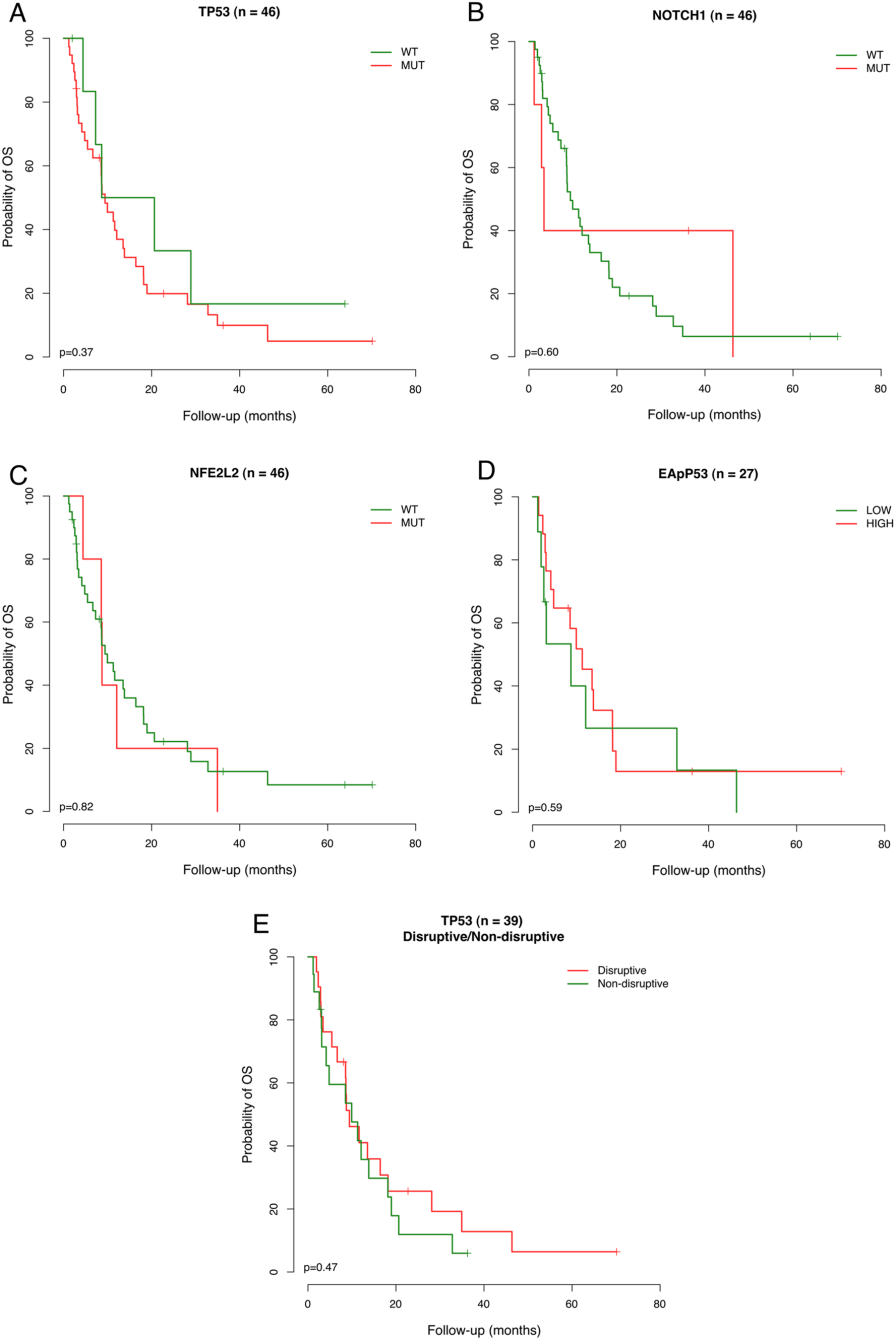
Kaplan–Meier curve for assessing the estimated overall survival probability of follow-up time among esophageal squamous cell carcinoma patients concerning mutation profile. A – *TP53* gene; B – *NOTCH1* gene; C- *NFE2L2* gene; D – Eap53 score; E- Disruptive and non-disruptive classification of TP53 mutations.

In addition, a Kaplan–Meier survival analysis was performed, and no association was observed with *TP53*, *NFE2L2,* and *NOTCH1,* neither with *TP53* EAp53 score and disruptive and non-disruptive categories (Table 5 and Fig. 3).

**Table 5:** Estimation of Global Survival by the Kaplan–Meier method considering patients’ time follow-up and mutation status and multivariable survival analysis (Cox regression model).

Variable	Category	Mean (months)	Overall survival	6 months	1 year	2 years	5 years	*p*-value
			95% confidence interval					
Global	–	9.40	6.101 to 12.715	65.5	38.8	21.9	7.3	–
*TP53*	MUT	9.40	6.282 to 12.534	62.5	36.9	19.9	5.0	0.373
	WT	8.65	0.000 to 24.681	83.3	50.0	33.3	16.7	
*NFE2L2*	MUT	8.71	8.364 to 9.070	80.0	20.0	20.0	0.0	0.822
	WT	9.40	5.555 to 13.261	63.3	41.6	22.2	8.4	
*NOTCH1*	MUT	3.42	2.220 to 4.622	40.0	40.0	40.0	0.0	0.627
	WT	9.40	5.570 to 13.246	68.7	38.5	19.3	6.4	
EAp53 status^a,b^	High	11.28	5.023 to 17.543	64.7	51.8	12.9	12.9	0.589
	Low	8.71	0.857 to 16.577	53.3	26.7	26.7	0.0	
TP53 disruptive/non-disruptive mutations^c^	Disruptive	9.40	5.169 to 13.647	66.7	41.0	19.2	6.4	0.497
	Non-disruptive	9.93	1.320 to 18.548	59.5	35.7	11.9	6.0	

*n* number of variants.

^a^ http://mammoth.bcm.tmc.edu/cgi-bin/panos/EAp53.cgi.

^b^Neskey et al. Evolutionary Action Score of *TP53* Identifies High-Risk Mutations Associated with Decreased Survival and Increased Distant Metastases in Head and Neck Cancer, Cancer Res. 2015 Apr 1;75(7):1527–36.

^c^Poeta et al. TP53 mutations and survival in squamous-cell carcinoma of the head and neck. N Engl J Med 357, 2552–2561, https://doi.org/10.1056/NEJMoa073770 (2007).

## Discussion

In the present study, we performed, for the first time, the somatic mutational profile of a panel of 150 cancer-related genes in a series of 46 Brazilian esophageal squamous cell carcinomas. We identified 25 genes with alterations, the genes with the highest frequencies being *TP53*, *NOTCH1*, and *NFE2L2*.

The frequency of mutation observed was compared with the literature, and similar frequencies were observed with other populations (Table 6). As expected, the *TP53* gene was the highest mutated (84.8%, 39 cases). In our study population, its frequency was similar to that reported in other studies such as TCGA^14–17,22–25^ (Table 6) and higher than previously reported in the Brazilian ESCC population (34–40%)^18,24,26^. This discrepancy with previous Brazilian studies is probably due to the distinct methodologies used since Sanger sequencing and hotspot *TP53* regions were used on those studies, at variance with all *TP53* coding regions and NGS used in the present study. We also applied for the first time a risk-system score based on Evolutionary Trace (ET) method proposed and validated in head and neck tumors (EAp53)^19^ to evaluate its impact in ESCC. We showed that 36% of *TP53* mutation in squamous cell carcinoma would be considered high-risk; however, there was no significant association between the clinical and pathological condition of the patient and the prognosis. This lack of significance could be related to the limited number of cases analyzed, and further studies are needed to validate our findings. We also classified *TP53* mutations in disruptive and non-disruptive, as reported by Poeta et al.^20^ and Molina et al.^21^, for head and neck and lung cancer, respectively. We observed that 54% were disruptive, yet no significant statistical association was observed with the clinical-pathological characteristics and patient prognosis.

**Table 6:** Comparison of mutation frequencies in esophageal squamous cell carcinoma patients.

Genes	Present study (n = 46)		TCG^a^ (n = 90)		CBioPortal (n = 227)		ICGC data portal (n = 332)		Sawada et al. 2016^b^ (n = 144)	
	N	(%)	n	(%)	n	(%)	n	(%)	n	(%)
*TP53*	39	(85.0)	83	(92.2)	156	(68.7)	244	(73.4)	134	(93.0)
*NFE2L2*	5	(11)	15	(16.7)	12	(5.3)	10	(3.0)	22	(15.9)
*CDKN2A*	3	(7.0)	3	(3.3)	8	(3.5)	19	(5.7)	11	(8.3)
*RB1*	3	(7.0)	4	(4.4)	18	(7.9)	12	(3.6)	6	(4.1)
*PTCH1*	1	(2.0)	8	(8.9)	6	(2.6)	15	(5.7)	5	(3.4)
*DDX3X*	1	(2.0)	1	(1.1)	–	–	6	(1.8)	2	(1.3)
*CDK12*	1	(2.0)	2	(2.2)	2	(0.9)	6	(1.8)	1	(0.6)
*BAP1*	1	(2.0)	–	–	3	(1.3)	8	(2.4)	2	(1.3)
*TERT*	1	(2.0)	2	(2.2)	1	(0.4)	2	(0.6)	2	(1.3)
*STAT3*	1	(2.0)	–	–	–	–	1	(0.3)	1	(0.6)
*NOTCH1*	5	(11.0)	13	(14.4)	19	(8.4)	47	(14.5)	27	(18.7)
*FBXW7*	1	(2.0)	3	(3.3)	8	(3.5)	15	(4.5)	8	(5.5)
*PTEN*	3	(7.0)	5	(5.6)	8	(3.5)	7	(2.1)	2	(1.3)
*PIK3CA*	2	(4.0)	12	(13.3)	15	(6.6)	33	(9.9)	15	(10.4)
*TSC2*	1	(2.0)	2	(2.2)	1	(0.4)	5	(1.5)	3	(2.0)
*BRAF*	1	(2.0)	2	(2.2)	2	(0.9)	4	(1.2)	2	(1.3)
*NF1*	1	(2.0)	2	(2.2)	8	(3.5)	11	(3.3)	4	(2.7)
*KRAS*	1	(2.0)	–	–	–	–	6	(1.8)	–	–
*MSH6*	1	(2.0)	2	(2.2)	3	(1.3)	3	(0.9)	3	(2.0)
*PALB2*	1	(2.0)	–	–	4	(1.8)	6	(1.8)	1	(0.6)
*KIT*	1	(2.0)	2	(2.2)	2	(0.9)	5	(1.5)	2	(1.3)
*ERBB2*	1	(2.0)	–	–	–	–	6	(1.8)	2	(1.3)
*RET*	1	(2.0)	2	(2.2)	–	–	1	(0.3)	1	(0.6)
*NT5C2*	1	(2.0)	–	–	1	(0.4)	2	(0.6)	1	(0.6)

cBioPortal for Cancer Genomics http://cbioportal.org; *ICGC* International Cancer Genome Consortium https://dcc.icgc.org/; *n* number of cases, *ESCC* esophageal squamous cell carcinoma.

^a^Cancer Genome Atlas Research et al. Integrated genomic characterization of oesophageal carcinoma. Nature 541, 169–175, https://doi.org/10.1038/nature20805 (2017).

^b^Sawada et al. Genomic Landscape of Esophageal Squamous Cell Carcinoma in a Japanese Population. Gastroenterology 150, 1171–1182, https://doi.org/10.1053/j.gastro.2016.01.035 (2016).

Another important gene of the cell cycle is the *CDKN2A* gene, also known as *p16,* which the loss of its function can result from homozygous deletions and mutations. According to the TCGA and genomic studies, the significant alteration is homozygous deletion, leading to 76% loss of function of the *CDKN2A* gene in ESCC^17^. At variance with high copy number alterations, *CDKN2A* mutation frequency is much lower, ranging from 3–8% (Table 6).

The second most mutated gene was *NOTCH1* (11%), which is part of the *NOTCH* signaling pathway, important to regulate the cell cycle senescence^27^. In esophageal squamous cell carcinoma, the frequency of mutations in this gene varies from 8 to 19% (Table 6), so our population is within the reported range. These findings are in concordance with the ESCC2 TCGA molecular subtype, observed in the 15 patients from South America (Brazil) analyzed^17^.

The *NFE2L2* gene encodes the NRF2 protein, a transcription factor regulating the expression of antioxidant proteins that protect against damage caused by injuries and inflammation^28^. Genetic deletion of NRF2 the susceptibility to the development of cancer increases, causing the tumor cell to survive the oxidative stress caused by chemoradiation leading to resistance to treatment^29–31^. The frequency of mutations in the *NRF2* gene has been reported in 3–17% of ESCC (Table 6). Driver mutations in *NRF2* are believed to be late events in the process of developing esophageal squamous cell carcinoma^22,32^. In our population, we observed a frequency of 10.8% of alterations in this gene, and in accordance with TCGA, it is associated with the ESCC1 subtype, typical of squamous carcinomas associated with tobacco exposure^17^.

The PI3K-AKT signaling is a complex pathway that regulates cell growth, proliferation, motility, apoptosis, and cell growth and is often related to the development of esophageal squamous cell carcinoma through driver mutations in the *PIK3CA* gene^15,16,33^. The frequency of mutations reported in the literature varied from 4 in the present to 13% (Table 6). Recently our group analyzed another series of 38 ESCC and observed a frequency of 10.5% of *PIK3CA* mutations^34^. The majority of the mutations are present in the hotspot exons 9 (E542K and E545K) and 20 (H1047R)^34–36^. (Supplementary Table S1). Another gene of this PI3K-AKT pathway is the *PTEN,* which is frequently mutated in some tumor types, such as melanomas and glioblastomas, yet its mutation rate in ESCC is lower, varying from 1 to 6.5%, as observed in our Brazilian series (Table 6). Of note, the TCGA ESCC3 subgroup is characterized by upregulation of this pathway, and in our study, it was alternated in 13% (*PTEN*, *PIK3CA*, and *TSC2)*.

The *KRAS* gene is an essential biomarker in cancer, mainly because it predicts the efficacy in therapies targeting the growth factor EGFR in tumors such as colorectal cancer^37,38^. According to the TCGA, the frequency of mutations in the *KRAS* gene is low, 7%, which is in line with the results obtained in our study population, which was 2%^17^.

ESCC is usually diagnosed late, and the minority of patients can benefit from treatments such as chemotherapy and radiation therapy^39^. Target therapies are important approaches for several tumors, including ESCC^39^. Significantly, in the present work, the identification of patients harboring *PIK3CA* mutations could potentially benefit from PI3K and mTOR inhibitors, such as buparlisib, alpelisib, and everolimus^40,41^. Moreover, the recent development of anti-*KRAS* agents, such as sotorasib and adagrasib^42,43^, can bring some hope for patients with *KRAS* mutations (Table 6).

The present study has several limitations, being the relatively small number of cases analyzed the major issue, and it could explain the lack of significant association of mutation status and patients’ clinical-pathological features. Additionally, a limited panel of 150 cancer-related genes, not whole-genome nor whole-exome sequencing, was performed, so a complete picture of the mutated landscape is lacking. Therefore, further studies with a larger population and broader mutation analysis are needed. Despite these issues, we performed paired germline/tumor analysis, and it is the first to the somatic landscape of an admixture population such as the Brazilian ESCC population. Our findings align with the frequencies reported in other populations, namely Occidental and Asian, and will contribute to understanding the mutational profile of esophageal squamous cell carcinoma in Brazil.

## Material and methods

### Tissue samples

Forty-six patients diagnosed with esophageal squamous cell carcinoma treated at the Barretos Cancer Hospital's upper-digestive department, Barretos, SP, Brazil, were evaluated. The main clinicopathological features were collected from patients' medical records.

The tumor and blood samples were obtained from biopsy or surgery and immediately processed and stored at − 80 °C in the Barretos Cancer Hospital Biobank. The present study was approved by the Barretos Cancer Hospital Institutional Review Board (Project No. 1.454.967/2016), and all patients included signed an Informed Consent Form. All methods were performed following the relevant guidelines and regulations.

### DNA isolation

Tumor DNA was isolated from fresh-frozen tissue using QIAsymphony DNA Mini Kit following the Tissue_200 protocol for automated isolation in the QIAsymphony (QIAGEN, Hilden, Germany). DNA from leukocytes of peripheral blood was isolated using the QIAmp DNA Blood Mini Kit (QIAGEN, Hilden, Germany), following the manufacturer's instructions. DNA quantity and quality were assessed by Qubit (Thermo Scientific, Wilmington, DE, USA).

### Mutation profile

The mutation profile of a commercial panel of 150 cancer-related genes was conducted at Mendelics Genetics company (São Paulo, SP, Brazil, https://www.mendelics.com/oncologia/) as previously reported^44^. The panel analyzed all coding sequence of the following genes: *ABL1, AKT1, AKT2, AKT3, ALK, APC, AR, ARAF, ATM, AURKA, AURKB, AXIN1, AXL, BAP1, BARD1, BCL2, BCL2L1, BCL2L2, BLM, BRAF, BRCA1, BRCA2, BRD2, BRD3, BRD4, BRDT, BRIP1, BTK, CBFB, CCND1, CCND2, CCND3, CCNE1, CD22, CD274, CD79A, CD79B, CDH1, CDK12, CDK4, CDKN1A, CDKN2A, CHEK1, CHEK2, CREBBP, CRKL, DDR2, DDX3X, EGFR, EPHA7, ERBB2, ERBB3, ERBB4, ESR1, EZH2, FAM175A, FAS, FBXW7, FGFR1, FGFR2, FGFR3, FGFR4, FHIT, FLT1, FLT3, FLT4, FRS2, GATA2, GNA11, GNAQ, HDAC1, HDAC4, HDAC7, HGF, HRAS, IDH1, IDH2, IGF1R, JAK1, JAK2, JAK3, KDR, KIT, KRAS, MAP2K1, MAP2K2, MAP2K4, MAP3K1, MAPK1, MCL1, MDM2, MET, MLH1, MPL, MRE11A, MS4A1, MSH2, MSH6, MTOR, MUTYH, MYC, MYD88, NBN, NF1, NF2, NFE2L2, NFKBIA, NOTCH1, NOTCH2, NOTCH3, NRAS, NT5C2, NTRK1, PALB2, PDGFRA, PDGFRB, PDK1, PIK3CA, PIK3CB, PIK3CG, PMS2, PTCH1, PTEN, RAC1, RAD51, RAF1, RANBP2, RARA, RB1, RET, RICTOR, ROS1, RRM1, RUNX1, SDHB, SMO, SOX2, SRC, STAT3, STAT5B, STK11, TERC, TERT, TGFBR2, TP53, TSC1, TSC2, VEGFA, WT1,* and *XPO1*. For sequencing, paired tumor and blood DNA libraries were prepared using Nextera Rapid Capture Custom Enrichment kit (Illumina, San Diego, CA, USA). Qubit Fluorometer quantified libraries, and Agilent 2100 Bioanalyzer evaluated their quality. The cluster generation and sequencing were performed in Illumina HiSeq 4000 following the manufacturer's instructions. Paired-end reads from Illumina sequencing were obtained by script bcl2fastq (v. 2.17.1.14), and data pre-processing was performed following the recommended best practices, i.e., alignment against the human genome reference build GRCh37 using Burrows-Wheeler Aligner (BWA, version 0.7.13), duplicates were marked, and a further base quality score recalibration step was applied^45^.

The VarScan2 algorithm called the somatic variants^46^. The variants with artifacts due to indel reads at their position, or less than 10% or more than 90% of variant supporting reads on one strand, were removed. The variants were further filtered to remove those with fewer than ten reads covering the variant and less than 5% of the variant allele frequency.

A second algorithm was applied using the somatic SNV and indell caller MuTect2 from GATK. MuTect2 combines the somatic genotyping engine of the original MuTect^47^ with the assembly-based machinery of HaplotypeCaller provided by GATK^48^, detecting somatic mutations using a Bayesian classifier approach. The variants were detected by comparing the likelihood of the site to be to sequencing noise and filtered by the alterations of the normal paired control (blood) from the same patient, by a pool of normals of 291 local samples using the same NGS technology and according to the allelic frequencies provided by the Genome Aggregation Database (gnomAD) datasets^49^ to reduce miscalled germline calls. In this study, only variants found by both variant callers (VarScan2 and MuTect2) were considered. Finally, the annotation of variants was done using Variant Effect Prediction^50^.

To identify driver mutations in tumors, we applied the Cancer Genome Interpreter-CGI^51^. Briefly, CGI annotates potential driver mutations detected in tumors by identifying known tumorigenic variants and classifying variants of unknown significance via OncodriveMUT^51^. After the CGI classification, we maintained variants classified as tumorigenic and variants predicted as Tier1 or Tier2 for tumorigenesis. Variants that were not classified as cancer driver mutation or not predicted as Tier1 or Tier2 as driver by the OncodriveMut algorithm were excluded. Therefore, mutations identified as polymorphism (high allele frequency) or predicted as neutral or passenger for oncogenesis and found in DNA sequence outside coding regions were excluded.

### Classification of *TP53* mutations

To assess the impact of *TP53* missense mutations, we used a risk-system score based on the Evolutionary Trace (ET) method proposed and validated in head and neck tumors by Neskey et al., called evolutionary action score of p53 (EAp53) (http://mammoth.bcm.tmc.edu/cgi-bin/panos/EAp53.cgi)^19^. In this system, mutations are scored from 0 to 100, with higher scores representing more deleterious mutations. A threshold score of 75 is used to classify variants as low-risk (EAp score < 75) or as high-risk (EAp score > 75). Mutations classified as high risk was associated with a poor prognosis, decreased survival, and increased development of distant metastases in head and neck tumors^19,52^.

Additionally, we evaluated a second classification proposed for head and neck tumors by Poeta et al.^20^ and validated in lung cancer by Molina et al.^21^. According to this system, *TP53* mutations are divided into “disruptive” and “non-disruptive”. Disruptive mutations are stop-codon all over the coding region and missense mutations within the L2 and L3 sites, codons 163–195 and 236–251 with an amino acid polarity shift. Non-disruptive mutations are missense mutations within the L2 and L3 sites and do not change in polarity between the amino acids.

### Statistical analysis

Characterization of the study population was analyzed through frequency tables for qualitative variables and measures of central tendency and dispersion (mean, standard deviation, minimum, and maximum) for the quantitative variables. Regarding the clinicopathological association analyzes with profile mutation (*TP53, NFE2L2,* and *NOTCH1*) and classification of *TP53* mutations (EAp53 score and disruptive and non-disruptive), we used the Mann–Whitney test for age, and for other categorical variables, the Chi-square test or Fisher's exact test.

The level of significance adopted was 5% (*p* ≤ 0.05). Statistical analyses were performed using the SPSS software v.21.0 (SPSS, Chicago, IL).

## Supplementary Information

Supplementary Information.

## Data Availability

Data that support the findings are available upon reasonable request and with the permission of Dr. Rui Manuel Reis.
